# Rock Climbing, Risk, and Recognition

**DOI:** 10.3389/fpsyg.2018.01793

**Published:** 2018-09-24

**Authors:** Tommy Langseth, Øyvind Salvesen

**Affiliations:** Department of Sports, Physical Education and Outdoor Studies, Faculty of Humanities, Sports and Educational Sciences, University of Southeast Norway, Bø, Norway

**Keywords:** risk-taking, Cred-Zone, extreme sport, recognition, Pierre Bourdieu, climbing

## Abstract

As extreme sports gain popularity – so does the public appreciation of such sports. Mass media are full of panegyric appraisals of these self-driven, individualistic athletes that dare to “live life to the fullest.” Voluntarily seeking risks, in general, and in extreme sports specifically, is often understood in terms of individual traits or the unique, strong emotions such experiences give. In this article, we move beyond individualistic explanations of risk-taking that understand risk-taking as personal traits. Instead, we focus on processes of recognition based on group values. More specifically, based on autoethnography and interviews with elite climbers in Norway, we explore to what extent risk-taking is built into the value system of climbing, and to what degree risk-taking leads to peer-recognition and credibility within rock climbing communities. We find that there is a clear connection between risk-taking and recognition in the value system of climbing. As newcomers become part of the climbing culture they learn what has value and make these values part of their own intrinsic motivation. Hence, climbers develop what we call a risk-libido. However, the results show that there are no clear-cut demarcations between actions that lead to recognition, actions that go unnoticed and actions that lack credibility because they are seen as foolhardy. The fact that these boundaries are not clear, does not mean that boundaries do not exist. Based on our findings, we develop and propose a model of “Credibility-Zones” that establish the general principles of honor- and status distribution within rock-climbing in regard to risk-taking. Of particular interest is our finding that among the most respected, “consecrated,” climbers, the “Credibility-Zone” is wider and less defined than for average climbers.

## Introduction

When climbers are forced to legitimize their risk-taking behavior, they will usually say something like “It’s fun,” “It gives me great experiences of and with nature,” or “It’s about mastering something difficult and potentially dangerous.” A philosophically inclined climber might answer with a quote from the Norwegian existentialist and climber Peter Wessel Zapffe: “Mountaineering is meaningless as life itself—therefore its magic can never die” (Zapffe, 1969/2012, p. 93). Or maybe they have given up explaining altogether and just respond, “Because it’s there.” In this article, we will give this a little twist and state that climbers do not climb because the mountain “is there” but because other people “are there,” that means that we will explore the social component of risk-taking behavior in regard to climbing.

Risk-taking behavior has puzzled researchers from various academic backgrounds for a long time. Considerable research has been conducted on themes such as risk-taking and personal psychological traits on the one hand and risk-taking and experiences, such as flow, on the other. However, little research has focused on the social psychological mechanisms behind risk-taking. To explain our approach to risk-taking, it is necessary to give a brief overview of the academic literature that describes and explains the phenomena of voluntary risk-taking. The different ways of understanding risk-taking, at least in regard to sports, can be broadly summarized with three main approaches: (1) the individualistic, (2) the “phenomenological,” and (3) the sociological.

What we call the individualistic approach is characterized by the understanding of risk-taking as something deeply personal. According to this approach, the propensity, or should we say “need,” to do dangerous things is deeply rooted in the individual. Some people are born to seek out dangerous situations. This might be because their genetic make-up makes them “high sensation seekers” or just because they are, for instance, type T personalities (e.g., Zuckerman, 1979, 2007; Farley, 1986; Breivik, 1996).

The “phenomenological” approach does not point at deeply rooted individual traits but at the deep emotions and experiences provided by involvement in sports that typically involve risk. “Phenomenological” is set in quotation marks here, because much of the literature we are aiming at is connected to Csikszentmihalyi’s (1991) flow theory, which, even if it is sometimes referred to as phenomenology, does not entail a rigorous phenomenology in either the concept’s philosophical or methodological sense. These theories focus on how actions that involve the right balance between challenge and skill are the source of emotions and feelings that are so satisfying that, to put it simply, no other explanation of why people do dangerous things is needed (Csikszentmihalyi, 1991; Jackson and Csikszentmihalyi, 1999).

The macro-oriented sociological approach seems to go in two directions, one compensation model and one adaptation model (Langseth, 2011). The compensation model understands participation in risk sports as a “safety valve” in a society that is overly concerned with safety. Risk sports, then, are a way of escaping a constraining, boring modernity. The adaptation model holds the opposite view, that increasing numbers of people turn to risk sports not because they are turning their backs on society but because the cultural norms of our modern society, the zeitgeist, demand that we live exiting, creative, autonomous, liberated lives. We ought to “seize the day”—and the day had better not be dull! According to the adaptation perspective, risk sports and their participants are not frowned upon; rather, the participants are a kind of cultural hero for our time. According to adaptationists, cultural imperatives in modernity push people toward risky sports.

The approaches above are certainly simplistically presented; there are more than three different ways to understand risk-taking. Nor are we suggesting that any of the above approaches are wrong or flawed. However, we need the above approaches to frame our own understanding of risk-taking. In this article, we argue that to understand “risk-libido,” the driving force behind risk-taking, we should neither merely study individual traits and experiences nor focus solely on macro-level social changes, but should also include social processes that make risk-taking logical and rational at a group (meso) level. Several studies have emphasized the importance of studying the context wherein risk rationalities develop (Hunt, 1995; Booth, 2003; Donnelly, 2004; Laurendeau, 2006; Beedie, 2007; Fletcher, 2008; Langseth, 2012, 2016). Based on the insight from these studies, this article focuses on the relational aspects of human behavior. That means that we are not studying individual climbers’ motivations and experiences but, rather, how established sets of values in the climbing community influence the individual climber. In our understanding, then, to study risk-taking in climbing, we must focus on what goes on between people in a group and not what goes on “inside” each individual. Booth and Thorpe (2007, p. 183) claim that in risk sports cultures, “…risk constitutes challenges, and meeting these challenges earn members—predominantly young men—rewards (e.g., peer recognition and prestige).” Similarly, Hunt, in a study of scuba divers, found that the divers “…are exposed to a competitive, informal system that places high status on the ability to dive deep-water wrecks and gather artifacts, both of which involve considerable risk” (Hunt, 1995, p. 445). In his study of base-jumpers, Langseth (2012, 2016) found that base-jumpers denied that risk-taking held any value in their culture, claiming that they base-jumped solely for their own sakes. Still, Langseth (2012, 2016) maintained that risk-taking was cultivated by subtle means. Langseth argues that risk-taking could be seen as a form of symbolic capital that gives rise to status and power hierarchies within the base-jumping community. The question, then, is whether we can find the same mechanisms within rock climbing. Robinson (2008, p. 149) states that to a degree, climbers take risks to achieve sporting success. In this study, we will explore and develop risk-taking along the same lines that Langseth (2012, 2016) did for base-jumpers. We will query into how group-level processes facilitate risk-taking by following three research questions. (1) What holds value in the field of rock climbing? (2) Is risk-taking a value in the climbing community? (3) What are the boundaries between actions that gain a climber recognition and credibility and actions that are deemed foolhardy?

The aim of the study is to get a better grasp of risk-taking by accounting for how subcultural values influences the actor. By doing this, we want to highlight the social aspects of risk taking. We are not saying that individual propensities and experiences are not important when it comes to understanding motives for risky actions. However, the social facets of risk taking is understudied. Hence, the importance of this study is that it expands the understanding of voluntary risk taking in sports.

## Materials and Methods

Methodologically, this article relies on two sources: qualitative interviews and autoethnography. To establish the values, hierarchies, and value hierarchies connected to risk-taking within the Norwegian climbing community, this study primarily depends on the authors’ own experiences in rock-climbing communities. One of the authors has been part of the Norwegian climbing community as both a climber and instructor since the early 1990s. The other author has been climbing since the mid-2000s and is currently qualifying as an international climbing guide through IFMGA (International Federation of Mountain Guides Associations). Even though our involvement with rock climbing does not qualify as systematic ethnography in the sense of rigorous participant observation, we still think that our participation puts us in a good position to outline the values that climbers adhere to. This places this project within the form of autoethnography that Anderson (2006) calls “opportunistic CMR” (complete member researcher), in which membership within a culture precedes the decision to do research on it. According to Ellis and Bochner (2006, p. 445) the term “autoethnography” should be reserved for research that “expresses fieldwork evocatively.” We do not agree. We would rather, in line with Anderson (2006), argue that autoethnography (and ethnography) should strive toward creating a theoretical understanding of the studied phenomena. The strength of autoethnography, as we see it, is that the researchers can have a full, embodied understanding of the culture under scrutiny while at the same time maintaining a critical distance from the participants’ meanings and statements about their motives, values, and goals.

Following Thorpe (2010), understanding participants’ world view and at the same time being critically distanced makes ethnography a hard task to master. The problem with “going native,” where the researcher becomes so engrossed that the distance from the researched culture disappears, is well described in ethnographic literature (e.g., Hammersley and Atkinson, 1996; Fangen, 2004). One could argue that the “going native” issue is even more problematic within autoethnography, where the researcher’s subjectivism and personal involvement is often thought to influence the outcome of the research. Hayano (1979, p. 101) points out that this is not necessarily a problem but is, rather, an asset that contributes to a deeper understanding. The risk aspect of climbing and its connectedness to credibility is not often discussed openly in the climbing culture. Our theme must be said to have a somewhat disguised character. Every climber tacitly knows that there is a connection between risk-taking and status, but a researcher without involvement with the climbing culture might very well run the risk of taking climbers’ statements at face value. So when climbers (or other extreme sports participants) state, “We don’t care about status,” “We do this solely for ourselves,” “The winner of the day is the guy with the biggest smile,” “Risk is something we avoid,” and so forth, researchers without climbing experience might end up taking these narratives as the truth. In other words, we argue that our involvement with climbing makes it possible for us to be more critical and more distanced than researchers who have not been part of the climbing culture. Our participation in the climbing culture makes it possible for us to recognize learned figures of speech—ways of speaking about motivation and so on that climbers learn as they are socialized into the climbing culture, ways of speaking that conceal from the speakers the real processes behind their actions. For instance, many climbers would state that rock climbing is an individualistic endeavor and that there are no status hierarchies in the sport. However, as climbers we know that there are distinct status hierarchies concerning who are considered good or solid climbers and that there are certain climbers and climbing styles that are held in high regard. This means, as mentioned, that the participants’ statements cannot be taken at face value. So when climbers talk about risk, risk-taking, and risk avoidance, we do not take these proclamations as the truth about what these themes mean for climbers. Rather, we take their statements as expressions of ways in which they have learned to talk about these themes within the climbing culture and the legitimate ways of speaking within the culture. In our view, the advantage of autoethnography is not that it makes “sensitive” presentations of the participants’ world view possible but rather, that it makes it possible to understand and at the same time be highly distanced from these world views. Being “indigenous” and being researchers, we think, puts us in a privileged position. The French sociologist Pierre Bourdieu (1999, p. 198) states:

*Social games are hard to describe in their double truth. Those who are captured by them barely have any interest in objectifying the game, and those who are not are often too poorly positioned to explore and experience what cannot be learned and understood without participating in the game.* (Our translation)

In our understanding, autoethnography, embodied in the researcher–climber, entails just this—the opportunity to understand the double truth of the climbing “game,” on the one hand understanding climbers and motives and on the other hand having the possibility of objectifying the same motives and feelings, tracing the discourses that have produced them. This means that we must somehow break with the agents’ own explanations. In Bourdieu’s words, we must perform an epistemological break. According to Bourdieu et al. (1991), it is important in empirical research to question and break with established truths and to reestablish actors’ statements within a theoretical framework. In practice, that means reinterpreting the actors’ actions and explanations in the light of the climbing field and its value system. This means that we, as researchers, and climbers will probably have diverging views on the truth about risk-taking in climbing. Our analytical understanding of climbers’ reality does not necessarily correspond to their own understanding.

In addition to autoethnography, and to avoid our own experiences from the climbing field being the only source of data, semi-structured interviews (Kvale and Brinkmann, 2009) with five elite Norwegian climbers were conducted to acquire information about climbers’ personal narratives of risk-taking. The interviews were conducted using an interview guide with certain predefined themes while at the same time being open enough to allow for follow-up questions and an elaboration of relevant themes that came up during the interviews. The predefined main themes in the interview guide were: climbing background, Motivation for climbing, socialization into the climbing culture, the climbers relationship to own risk taking, the climbers understanding of other climbers’ risk taking and their understanding of hierarchies within the climbing community. All interviews were fully transcribed. The interviewees were selected based on certain criteria. We sought climbers with a strong connection to and high standing within the Norwegian field of climbing. We also wanted climbers who were heavily involved with ice climbing, alpine climbing, and/or climbing with natural protection. The reason for this is that the risk involved in these forms of climbing is more prominent than in more popular forms of climbing, such as bouldering or sports climbing (Schöffl et al., 2010). We also sought interviewees who were well known in the Norwegian climbing community, on the assumption that well-known climbers are looked up to by other climbers, and hence, they have some kind of power of definition. To find the right climbers, we went through all volumes of the Norwegian climbing magazines *Norsk Klatring*^1^ (Norwegian Climbing) and *Tidsskrift for Norsk Alpinklatring*^2^ (Journal of Norwegian Alpine Climbing) from 2008 until 2014 and contacted some of the most profiled climbers. We ended up with five climbers who are all well-known within the Norwegian climbing community. The youngest was in his early twenties and the oldest in his late forties. All of them were men. We do not have data on what percent of the Norwegian climbing population is female, but data from Great Britain suggests that 70% of British climbers are men (Llewellyn et al., 2008). It is our impression that even fewer women participate in the form of climbing activities that we sought after. A few Norwegian women fitted the types of climbers we were looking for, but unfortunately, we did not succeed in getting interview appointments with them. Former research in comparable activities reveals similar difficulties (Robinson, 1985; Slanger and Rudestam, 1997; Holland-Smith and Olivier, 2013). How the gender imbalance influences our result is hard to tell. Men are overrepresented in all outdoor life accidents in Norway (Horgen, 2013), which might indicate gender differences in behavior patterns as well as different forms of risk logics.

When analyzing the interviews, we held in mind Bourdieu’s view that an epistemology of continuity is mistaken (Bourdieu et al., 1991). Following Bourdieu, a true (social) science cannot be satisfied with presenting actors’ self-understanding but must grasp both the structure that influences actions and the structures behind the naïve representations of action. Bourdieu particularly criticizes phenomenological approaches for being naïve (Bourdieu et al., 1991); first of all, actors’ *experiences* are constructed from historical and social circumstances. Secondly, when actors are asked to talk about (self-interpret) their actions and experiences, the researcher will get answers that are historical and that are social constructions at a second degree, in other words, ways of speaking about things that are within a social structure. The researcher’s interpretation, then, will be a third-degree interpretation, far from the phenomenological “Sache selbsts.” Hence, in analyzing the transcribed material, our approach was what Kvale and Brinkmann (2009) call a *theoretically informed reading*. This involves reading through and analyzing the material with certain theoretical preferences in mind. The material has thereby undergone a type of meaning interpretation that seeks to uncover structures and meaning dimensions rather than what is directly said in the interviews. More inductively inclined researchers often criticize such theoretically informed analysis. It is often pointed out that the researcher will find nothing more than he or she knew before and will find only data that “fits” the theory. This might be a problem. However, as we see it, the presence of data that supports the theoretical prenotions simply indicates that the relationships we are looking for exist.

## Theoretical Approach

To understand how actors develop a desire to engage in risky actions, this article builds on the theories of Pierre Bourdieu (1977, 1993; 1999, 2004) and, to a certain extent, George Herbert Mead (1934). Bourdieu’s theories have been widely used to describe the relationship between social background and participation in sports (Laberge and Kay, 2002). However, in this article, we use Bourdieu in another way. We are interested in the cultural learning that takes place within the frames of rock climbing and in how this is connected to risk-taking. The connection between risk-taking and cultural learning in relation to windsurfing is made clear by Dant and Wheaton (2007): “…the physical capital of windsurfing, while it may give the sport its distinctive and extreme character, only becomes meaningful to participants, through the social process of a subculture (p. 11). So to understand risk-taking, we must look at how it is connected to the value system of particular sports subcultures.

Following Bourdieu, to understand how people act, we must understand the relation between social fields, forms of capital, and agents’ habitus. Even though we are not going to use the concept of habitus in this article, we have to say a few words about it to make Bourdieu’s theories coherent. Habitus can be seen as each individual’s “frame of action,” bodily ingrained experiences, and memories that determine how the person acts and understands the world. Habitus works below the threshold of language and makes the world appear in certain ways. The individual’s social background as well as other social groups that he or she has belonged to give roots to lasting habits and dispositions that determine how we think, feel, act, and dream about achieving (Bourdieu, 1977, 2004). Since every person has an individual biography, every habitus is unique. Still, habitus can be understood at a group level. Without delving into a discussion on philosophical agency, the point here is not that agents do not have some form of individual will but rather that they are, in one way or another, connected to and influenced by social relations. The climbers interviewed for this project have been through years-long socialization processes that produced in them a similar habitus; to some extent at least, they share the same values, dreams, and goals. It is important to note that habitus is in continual change; even though it might be resilient, it is not set once and for all after childhood but continues to evolve as persons get involved in new social fields.

In Bourdieu’s understanding, fields are social arenas where agents fight over symbolic or material interests that are common to them and only to them (Broady, 1991). Every field has its own norms and logic that agents must incorporate to be able to “play the game.” Bourdieu often uses the “game” metaphor to emphasize the element of competition, the presence of indisputable rules (which Bourdieu calls “doxa”), specific forms of investment, and rewards in every field (Bourdieu and Waquant, 1992, pp. 97–98). A central point is that the agents’ conscious knowledge of the rules of the game is minimal. The rules are taken for granted or seen as “natural.” The “naturalization” of these rules is what makes an investment in a field meaningful, at least in such a way that the meaningfulness of participating in a certain activity is not questioned. However, defining climbing as a field is not unproblematic. The concept was originally used to describe rather broad societal areas such as the political field or the field of literature. Compared to these fields, climbing is a rather narrow field. Still, it is not hard to legitimize the choice to see climbing as a field. Climbers share a common logic and a set of values that is generally tacitly accepted by most climbers. The practice around free ascents, where the climber must adhere to strict rules before being able to claim that she or he has climbed the route, illustrates this. Even though such rules are mostly taken for granted, they are a result of power struggles within the climbing field. Continuous power struggles have formed and are forming the field of climbing, for example, with respect to what is seen as “good style” (more about that later). The point is that as an aspiring climber is socialized and becomes a part of the climbing culture, the person also makes the rules and values that exist in the field a part of her or his own way of thinking and behaving, and the objective values of the field become internalized in each climber. Following Bourdieu (1993), determining what a field is, is the same as determining the forms of capital within a social arena.

Bourdieu is probably most known for the concept of cultural capital, a concept that highlights how knowledge of traditional “high culture” is related to power in a country such as France. In this article, we rely on his general concept of symbolic capital, which involves values that give recognition and prestige within a field. Symbolic capital is always related to a field, because without agents that understand an action, an artifact, or something similar as valuable, it would be worthless (Broady, 1991). A specific form of symbolic capital, let’s say economic capital, might very well be valued in several fields, but it can never be independent from fields. Money has no value in societies that do not use a monetary system. Training hard to free climb a certain climbing route would be worthless if not for the climbing field and the agents who have internalized the values of the climbing field, who recognize the activity of climbing as meaningful. To climb a long, demanding, difficult big wall route is only meaningful if there are other people who recognize the act as meaningful. Beal and Wilson (2004, p. 46) argue that extreme sports place risk-taking at center stage and that these activities “rely on risk and difficulty to determine status.” In this article, we explore whether and to what extent risk-taking can be seen as a form of symbolic capital within the field of climbing.

Bourdieu is often criticized for not explaining and not emphasizing how the actual process of socialization works. Several of our interviewees started climbing as adults, so perspectives on how they acquire the mental structures of the climbing field need a few more words. The symbolic interactionist George Herbert Mead’s theories about the development of the social self might suffice. According to Mead (1934), it is through interaction with other people that we develop a “self.” Mead’s self is a self that has learned and incorporated values and ideas from interaction with other people; it is a social self. The way we act and think is influenced by the expectations of other people. However, what we think is expected from us is dependent upon the specific situation. In close relation to Mead’s theories—even though it was not developed by him—is the concept of significant others. The concept describes persons in our primary network who are of special importance to us. This perspective is relevant to us, because it contributes a theoretical perspective on how climbers mirror themselves in other people’s reaction to their own actions and also why the meaning bestowed by some people might mean more than that bestowed by others. As the Norwegian climbing historian Geir Grimeland (2004, p. 236) points out, “It is the connoisseur’s recognizing nod which matters in the social life of the climber.” In other words, credibility in climbing is often allocated by subtle means rather than in clearly specified ways. That is, the rules are quite clear and specific, but they are also tacit, more often than not.

## Results and Discussion

### Risk-Taking and the Value System of Climbing

When climbers and other risk sports participants are asked about risk-taking, they are usually quick to state that they take all precautions to make the activity as safe as possible and that risk taking is not part of their motivation (see Brymer, 2010; Frühauf et al., 2017). Langseth (2012) found that base-jumpers often stated that they do not care about recognition and that they participate in the sport just because it is what they themselves want. Being part of the Norwegian climbing community, we have often heard similar utterances from climbers. But can we trust the explanations the climbers and other risk sport participants give? Not necessarily. First of all, climbers seldom have an articulate understanding of the values, rules, and logic of the climbing subculture. Secondly, when asked about motivation, they will give answers in a language that is shaped within the same subculture; they speak about motivation for climbing in the ways they have learned to talk about it within the climbing culture. That means that they will answer in the ways that are legitimate for a climber to speak about motivation. At the same time that the neophyte climber learns a new vocabulary for speaking about rock types, moves, and climbing gear, they also learn to speak about experiences and motivation. So when a researcher asks a climber about why they climb, their relation to risk-taking, etc., the answers are mostly prefabricated readymades. Thus, to truly understand the mechanisms behind risk-taking, we must look beyond the individual climbers’ statements. In society in general, but even more so in extreme sports, it is the individualistic explanations of action that are legitimate. “Intrinsic motivation,” where the agent is “engaging in an activity for itself and for the pleasure and satisfaction derived from participation” (Vallerand, 2004, p. 427), seems to be the only legitimate motive. A climber accepting that recognition is a driving force behind her or his action would be accepting the motivation does not come from “within.” Still, from Mead to modern neuroscience, we know that human beings are not autonomous entities—sociality is a basic human feature. We get our personal values and emotions by mirroring ourselves in other people (van Kleef et al., 2016). We learn to know ourselves by the feedback we get from our peers. Recognition, according to Bourdieu, is a driving force in human beings’ desires (Crossley, 2001). Thus, the values in a field – what gives recognition in a field – are connected to the individual actors’ motives. However, what provides recognition will vary from culture to culture, between social classes, and from subculture to subculture. Before we can say anything about the individual climbers’ motives in undertaking dangerous endeavors, we must lay out the value system of climbing and study the role that risk-taking plays within this system.

A novice climber will quickly learn the style hierarchy of climbing and that often in the climbing field, how something is done is considered more important than what is done. A climber’s approach, tactics, and choice of equipment on a climb are some of the factors determining what is often referred to as “style.” Beginners attending a typical 2-day introductory climbing course, which both authors have held multiple times over several years, are being taught early the all-important difference between “top roping” and “leading” a route. “Top roping,” with the rope fixed from above, is the safer, less committed way of climbing, whereas “leading” involves bringing the rope from the ground and clipping protection as you progress up the route, risking longer falls. The novice will learn that top roping is not considered a valid ascent. This means that already, from the first day of a climbing course, the novice learns that the riskier way of climbing holds higher value. The beginner will also learn that the chosen method of protection is of importance. Placing your own gear is considered far better than relying on pre-placed bolts. And climbing a poorly protected route brings more recognition than climbing a well-protected route of the same difficulty.

**FIGURE 1:**
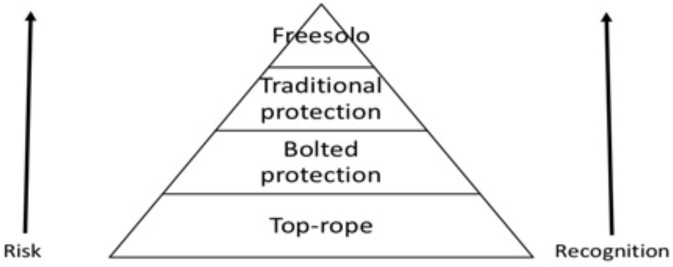
Style distinctions in free climbing.

The greatest and most recognized style of all, however, is using no ropes or protection gear whatsoever. American climber Alex Honnold’s free solo ascent of El Capitan in Yosemite in 2017 is a fine example of the massive credibility and public appeal this can generate. Most climbers would agree with this style hierarchy of free climbing, even though they are not involved in the activities at the top, and this is the same kind of hierarchy you will find in other branches of climbing. In alpine climbing, the so-called “alpine style,” where the climbers carry all their gear to the top in one push, is considered better the “capsule style” and “siege tactics,” which are, again, considered a better style than that of commercial expedition climbing. These styles are all values held by the climbing community, and can be seen as forms of symbolic capital. The wily thing about these hierarchies is that the actions at the top not only entail more recognition but are also more dangerous. The logic that the beginner quickly learns is that the more risk is involved, the more recognition and credibility a climber will get. This connection between risk and recognition is not usually directly spoken about and climbers are not necessarily aware of the connection between risk and recognition. That risk-taking holds value is hidden in terms such as “bold” climbing and “style.” Now, we are not saying that risk-taking is the only way to achieve status within climbing. There is no doubt that a sport climber such as Chris Sharma, mostly known for climbing hard but rather safe routes, has high status within the climbing community. Certainly, there are many ways for climbers to climb the hierarchy of climbing. But still, the risk-recognition nexus is taught under the disguise of “style” and “ethics” in climbing courses and introduction books (see for example Fyffe and Peter, 1990) (**Figure 1**). And as the novice becomes part of the climbing community, the risk-recognition logic is subtly communicated through rumors about climber X, who climbed that big wall route unroped, and climber Y, who only placed four pieces of protection on a hard pitch—conversations between friends around the campfire, which communicate values and convey the value of risk. Consider this text message that one of the authors received a while back: “Did a first ascent at Dale today. The route was exciting…” There is nothing extraordinary about this text message, but it says something important about the value logic of climbing. Let’s say the route was a French grade 7b and was naturally protected. That would give the climber recognition and credibility, or “cred” as we call it in order to stay more in touch with the language climbers use. The extra information that the route was exciting probably meant that it was poorly protected. The first ascensionist can thereby cash in even more cred. Throughout the disciplines of climbing, from small boulders to the tallest mountains on Earth, the logic of risk resulting in higher recognition is consistent, even though climbers are not usually consciously aware of this connection. We say “not usually,” because they might very well be. One of our informants, “Trym,” says:

If you look at me as a sports climber that is not such a good sports climber anymore and doesn’t send hard routes anymore… (I) have to, in a way, seek out the dangerous routes to get recognition in the community.

Risk-taking can thereby be seen as a field-specific form of what Bourdieu calls symbolic capital, a form of capital that leads to or can lead to increased status within the climbing field. The “risk capital” can, in some (rare) cases, even be converted into economic capital. Consider the American climber Kevin Jorgeson. When he came to fame in the climbing world, he was a boulderer. However, at the time he became well-known, he was not among the absolute top boulderers in the world. Rather, he became famous for doing “high-ball” boulder problems, boulder problems that top out so high above the ground that a fall would be dangerous. He was soon sponsored by Adidas and Duracell. This shows how risk-taking as a form of symbolic capital can be converted to economic capital.

There are elements of risk in most sports and outdoor activities. The question is whether or not risk is seen as problematic. The central point is that as people are socialized into the climbing culture, they internalize the field-specific values of that climbing culture. They learn “the rules of the game,” and slowly these rules become natural and unquestioned, to such an extent that they are seen as naturally given. The climbers start thinking that it is just natural that leading gives more cred than top-roping and that leading a poorly protected route is better than leading a well-protected one. They develop what Bourdieu (1996) calls a “sense of the game,” which means that the climbers become mentally structured in accordance with the logic of the game and forget that these rules are arbitrary. This gives them the feeling that the game is worth playing and worth investing in—and also that taking risks is valuable, because it is part of the game’s rules that risk-taking provides credibility. The arbitrariness of such rules is described by Bourdieu in this way:

*…What is at stake only exists for those who are involved in the game and are disposed to acknowledge the stakes, are ready to die for it—for something that seems entirely without interest from the perspective of those who are not involved in the game…*(Bourdieu, 1996, p. 134) [our translation].

Here, Bourdieu talks about general investments in all fields. But for climbers, being “ready to die” for something that seems worthless to outsiders is not a metaphor. The point is that even though the actions that climbers (and other extreme sports athletes) perform might seem spectacular, the mechanisms behind them are highly ordinary, involving learning and internalizing the values of a specific field or culture. The difference between climbing and other social games, let’s say stamp collecting, is that the internalized values are (at least partly) connected to risk and that they thereby develop what we might call a risk libido.

### The “Cred-Zone”

Now, is it all about risk, then? Does taking risks automatically entail a heightened status within the climbing community? Obviously, it is not that easy. If it were, any hazardous climber could attain instant legend status if he or she managed to stay alive for some time. Of course, there is more to this, and there are mechanisms to keep climbers from progressing too quickly in this field. Our experience from the field and interviews shows that there are a number of criteria that need to be fulfilled for risky behavior to receive credibility within the field.

Firstly, it has to be the right kind of risk. Voluntarily seeking out and climbing in avalanche terrain or ice climbing on poor ice because of the increased risk involved would not bring the climber recognition. The risk must be relevant, which means that it must be a form of risk that can be managed. Also, risking one’s life by purposefully placing bad protection or rigging a poor belay, although this would significantly increase the risk of what you were doing, would not increase your accumulation of symbolic capital. On the other hand, keeping a cool head while climbing a hard, poorly protected pitch, an action central in the climbing game, would likely give a climber status.

Secondly, and more importantly, having a margin of error seems to be important for a climb to be given recognition and. As one of our informants, “Rolf,” says:

…The important thing for me is that things are done in good style. Meaning that a climb is done in the way I think it should be done … in style and with margins. If you see that you don’t have margins, you should go back down.

The quote reveals that there are boundaries to the risk-recognition logic. This means that a climber must have margins in terms of a skill set and experience level that correlate to the climbing task at hand. If risk-taking is seen as being too much a game of chance, it seems like recognition is hard to attain. Margin is valued to the extent that our responders often expressed the idea that margin was synonymous with good climbing style. This trait was also previously documented by West and Allin (2010). It means that the skill level of the climber is important in the process of allocating status and prestige within climbing. For a climber to gain credibility in relation to risk, there must be the right combination of skill and risk involved. When talking about a friend of his that had just climbed a hard, scarcely protected route, “Trym” said:

You know, climbing that route is sick (…), but then I think that if he feels strong enough to climb it, then I actually thinks it is ok.

**FIGURE 2:**
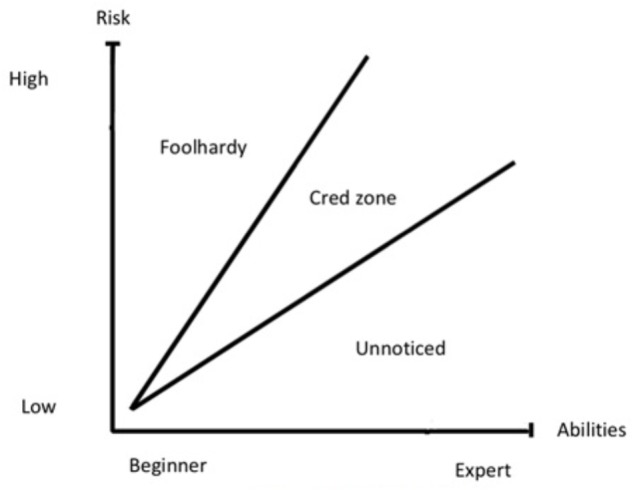
Cred-Zone model.

The logic here seems to be that *to feel strong* indicates that a climber has margins enough on a route to get credibility for climbing it. Even though the quote reveals that “margins” is highly individualized, it still means that there are some kind of boundaries between actions that are deemed credible and not, even if these boundaries are blurred. The link between skillset, risk and recognition has lead us to develop a generalized “Credibility-Zone” model (**Figure 2**). The model is inspired by Csikszentmihalyi’s “flow channel,” where the feeling of flow is dependent upon the right degree of challenge on one side and the skill level of the performer on the other side (1992). Our model is what we might call a sociological ideal type (Weber, 1978). That is, it is a simplification where typical traits of a social phenomenon are highlighted.

The model simply shows that to get “cred” for an action, you must have the right balance of risk-taking and skills. A beginner taking on a hard, bold route would usually not get recognition. This action would instead be deemed foolhardy. The same goes for experienced climbers. When talking about alpine climbing in alpine style, one of the informants, “Jarle” says:

There has been a lot of pushing it in Alaska and Patagonia that has been, well…where they have pushed it far. (…) We have discussed a couple of the ascents. Ascents that has been successful and has been given a lot of recognition, but I, and many others think that they have taken to many chances to achieve it. (…) I think there is a delicate balance, (…) there are things that are done in the bigger mountain ranges in very light styles… (…) it is not everything done that I think is cool…when you get a feeling that they took a lot of chances and pushed it too far…I don’t like that…when it is coincidence that determines if people survive…

The quote indicates that “pushing it to far,” even if the ascent is successful, does not necessarily give “cred.” The quote also reveals, as mentioned, that these boundaries are blurred, some climbers would give recognition and some would deem it foolhardy. The same blurred boundaries can be found in freeride skiing for instance. In 2006, Canadian skier Jamie Pierre set a new world record when he dropped an 82 m cliff. He landed on his head, but survived. In the discussion board of Norwegian skiing magazine “Fri Flyt” some commentators thought that this was “awesome,” while others saw this as an stupid act that should not be given any recognition^3^. Again, this means that there are no clear-cut line between risky acts that are given recognition and those deemed foolhardy, but that does not mean that there are no boundaries.

On the other side of our model is actions that does not get recognition because the acts are too quotidian. An experienced climber, climbing an easy, safe route, would not get much cred because the action would likely go unnoticed. For an experienced climber, climbing a four pitch well protected 5.10 would not raise his or her status. In other words, climbing a safe, easy route, would not earn an accomplished climber (more) symbolic capital than what she or he has from before. For a relatively new climber, however, climbing the same route would give credibility. Over the course of our work, we realized that some climbers’ achievements were not met with the same criticism and skeptical evaluation of their efforts as those of other climbers were. There was a clear pattern where more experienced climbers were enjoying the benefit of the doubt to a far greater extent than less experienced ones. One informant, “Sander,” said that

There is a quite definite borderline between experienced and inexperienced climbers. When you have passed that line it is more accepted that you take calculated risks.

Therefore, the Cred-Zone in **Figure 2** is shaped like a funnel. In general, beginners seems to have fewer opportunities to earn cred; they have not yet invested enough in the field. On the other hand, experienced climbers seems to have more “slack” regarding what is deemed foolhardy or not. In the quote from “Trym” above an experienced climber is given cred because “*he feels strong enough.*” Our experience from the climbing field is that beginners are not given the same leeway when it comes to what gives cred. This, however, is an assertion that needs more empirical data before anything definite can be said about the shape of the Cred-Zone.

There also seems to be a line between experienced climbers and climbers that almost have a “hallowed” status that expands the boundaries for what is given cred. When talking about one of the top Norwegian climbers known for his ascents in the big wall Troll Wall in Norway, “Ola” said:

By climbing Troll Wall that many times in a row there is a clear increased risk for something to happen (…). I absolutely think that it is really cool that he is pushing it.

In Bourdieusian terms, we can say that some climbers might be considered “consecrated” (Bourdieu, 2004). That means that they are not just seen as experienced climbers, but they also achieve an almost sanctified insider status where their actions cease to be scrutinized in a critical manner. When talking about a climber who had just done a first free ascent of a scarcely protected route at Troll Wall, “Rolf” was very impressed and said:

It is obvious that what NN is doing, performance wise, is at a very high level. I have no idea about what style he is doing it in, in regard to risk. I just see that he is taking things a step further, (…) but I have no idea about how he does it.

**FIGURE 3:**
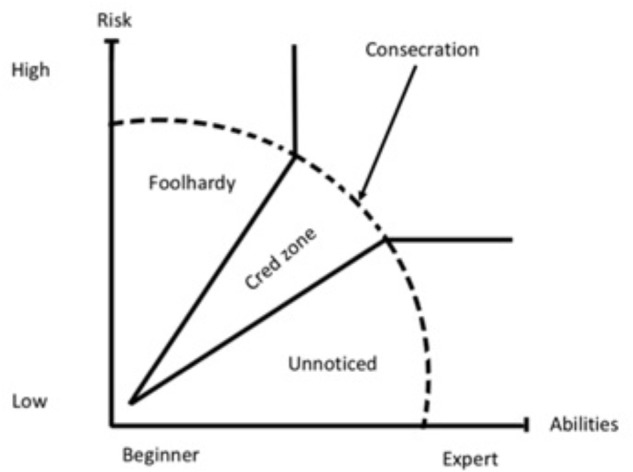
Cred-Zone and consecration.

So, for this highly regarded climber the previous mentioned importance of “style” is not longer important. He still get cred, even though “Rolf” says he no idea about how the route was climbed. Exactly where the line between the consecrated and the unconsecrated is drawn is not easy to say, but it has to do with the accumulation of symbolic capital. In free climbing, it might be climbing a certain grade, whereas in alpine climbing, where the difficulties are more complex, having climbed certain routes might symbolize this transition. The interesting thing for us was that this led us to a slight change to the general Cred-Zone model. As shown in **Figure 3**, the Cred-Zone expands after consecration. That means that after consecration, it is easier to gain credibility, as an act is not seen as foolhardy by the same criteria as for other experienced climbers.

## Conclusion

A common objection from climber friends when we talk about this project is that their motives for climbing have nothing to do with recognition or status. “It’s all about having the feeling of mastering something; mastering something difficult is what gives us good experiences, which also means mastering stuff that is a bit scary and dangerous,” they typically say. We agree. But what is it that climbers wish to master? What they wish to master is not something they themselves have defined. Climbing is not idiosyncratic. Rather, what climbers strive for, the routes they have a burning inner desire to fulfill, the mountain they want to summit because it is there, the lovely feeling of pulling off that move or placing that perfect cam—it all stems from their relation to the subculture of climbing. The central point is that what has value in the subculture of climbing is made personal; the values are internalized and made the climber’s own values. This means that what is experienced as intrinsic motivation is connected to the recognized values in the climbing field or subculture. In other words, internal motivation is external, or has been external, in the sense that they stem from values that existed within the climbing culture prior to the individual climber entering it. This does not mean that climbers consciously act to get attention and recognition. Some do, of course; the use of recognition technology like Instagram or Facebook leaves no doubt about that. However, our point is that as climbers are socialized into the climbing culture, the values of this culture become part of their own inner life. What holds value and gives recognition within the climbing culture and becomes part of the individual climbers’ own goals and dreams. From this perspective, climbers strive after what gives recognition, even though no one else would even know that they have climbed something recognizable.

The contribution of this article has been to establish the often-overlooked relationship between risk and recognition in climbing. By not taking climbers’ statements at face value but instead highlighting the style hierarchies of climbing, we have argued that climbers develop a risk libido, a drive toward risk-taking, as part of being immersed in the climbing community. Yet we have also pointed out that risk, recognition, and status are not solely about doing dangerous climbs. To cash in on the symbolic capital that risk-taking is, certain considerations must be taken into account. An act deemed foolhardy by other climbers will not grant any status. The climber must show that they are in control and have margins on what they are doing. They must also show that there is correspondence between their skills and the risk they are taking. On the other side, doing a climb that is considered too easy or safe for skilled climber would not bring recognition either. Thus, in conclusion, to climb the hierarchy of climbing, you need balance—an embodied kind of balance that only a fine-tuned sense of the climbing game can give.

An interesting task for future research would be to test and further develop the Cred-Zone model in relation to other extreme sports. The model should also be tested by quantitative research, that could more clearly and precisely draw the lines between what is recognized and what is deemed foolhardy in an extreme sport culture.

## Ethics Statement

This study was carried out in accordance to the requirements of NESH, the Norwegian National Committee for research in the Social Sciences and the Humanities. All participants received written information concerning informed consent before agreeing to be interviewed.

## Author Contributions

Both authors have made substantial contribution to the work and approved it for publication.

## Conflict of Interest Statement

The authors declare that the research was conducted in the absence of any commercial or financial relationships that could be construed as a potential conflict of interest.

## Footnotes

1 Six issues per year.

2 One issue per year.

3 https://www.friflyt.no/Ski/Jamie-Pierre-med-ny-verdensrekord-82-meter/Diskusjon-av-artikkel-Jamie-Pierre-med-ny-verdensrekord-82-meter
